# Sensor-based postural feedback is more effective than conventional feedback to improve lumbopelvic movement control in patients with chronic low back pain: a randomised controlled trial

**DOI:** 10.1186/s12984-018-0423-6

**Published:** 2018-09-26

**Authors:** Thomas Matheve, Simon Brumagne, Christophe Demoulin, Annick Timmermans

**Affiliations:** 1 Rehabilitation Research Center - Biomed, Faculty of Medicine and Life Sciences, Hasselt University, Hasselt, Belgium; 2 Department of Rehabilitation Sciences, KU Leuven – University of Leuven, Leuven, Belgium; 3 Department of Sport and Rehabilitation Sciences, University of Liege, Liege, Belgium

**Keywords:** Low back pain, Feedback, Movement control, Motor learning, Sensors, Technology

## Abstract

**Background:**

Improving movement control can be an important treatment goal for patients with chronic low back pain (CLBP). Although external feedback is essential when learning new movement skills, many aspects of feedback provision in patients with CLBP remain currently unexplored. New rehabilitation technologies, such as movement sensors, are able to provide reliable and accurate feedback. As such, they might be more effective than conventional feedback for improving movement control. The aims of this study were (1) to assess whether sensor-based feedback is more effective to improve lumbopelvic movement control compared to feedback from a mirror or no feedback in patients with chronic low back pain (CLBP), and (2) to evaluate whether patients with CLBP are equally capable of improving lumbopelvic movement control compared to healthy persons.

**Methods:**

Fifty-four healthy participants and 54 patients with chronic non-specific LBP were recruited. Both participant groups were randomised into three subgroups. During a single exercise session, subgroups practised a lumbopelvic movement control task while receiving a different type of feedback, i.e. feedback from movement sensors, from a mirror or no feedback (=control group). Kinematic measurements of the lumbar spine and hip were obtained at baseline, during and immediately after the intervention to evaluate the improvements in movement control on the practised task (assessment of performance) and on a transfer task (assessment of motor learning).

**Results:**

Sensor-based feedback was more effective than feedback from a mirror (*p* < 0.0001) and no feedback (*p* < 0.0001) to improve lumbopelvic movement control performance (Sensor vs. Mirror estimated difference 9.9° (95% CI 6.1°-13.7°), Sensor vs. Control estimated difference 10.6° (95% CI 6.8°-14.3°)) and motor learning (Sensor vs. Mirror estimated difference 7.2° (95% CI 3.8°-10.6°), Sensor vs. Control estimated difference 6.9° (95% CI 3.5°-10.2°)). Patients with CLBP were equally capable of improving lumbopelvic movement control compared to healthy persons.

**Conclusions:**

Sensor-based feedback is an effective means to improve lumbopelvic movement control in patients with CLBP. Future research should focus on the long-term retention effects of sensor-based feedback.

**Trial registration:**

clinicaltrials.gov NCT02773160, (retrospectively registered on May 16th, 2016).

**Electronic supplementary material:**

The online version of this article (10.1186/s12984-018-0423-6) contains supplementary material, which is available to authorized users.

## Background

The lifetime prevalence of low back pain (LBP) is reported to be as high as 84%, whereas the estimated prevalence of chronic LBP (CLBP) is approximately 23% [1]. Globally, it is the leading cause of disability [2] and one of the most important reasons for work absenteeism, resulting in a high socioeconomic burden [3].

Patients with CLBP form a heterogeneous group, which is exemplified by the differences in movement patterns within this population. While some patients with CLBP stiffen their spine and avoid spinal movements, others show the opposite pattern and adopt end range postures or move excessively into their painful direction [4]. For the latter type of patients, movement control exercises are often prescribed [5]. The aim of these exercises is to learn how to control movements into the painful direction, thereby reducing the mechanical load on the painful structures and decreasing peripheral nociceptive input [6].

Changing movement patterns requires motor learning. The importance of external feedback (i.e. feedback coming from a source external to the person performing the task [7]) in motor learning has been well established, and optimizing the way feedback is provided is therefore essential [8, 9]. While there is an abundance of literature on the role of extrinsic feedback to improve motor learning in a healthy population, many aspects of feedback provision in patients with LBP remain currently unexplored [9]. When patients with LBP perform lumbar movement control exercises in the absence of a therapist, they typically have to rely on visual feedback (e.g. from a mirror) or palpation [10]. However, the reliability and accuracy of these types of feedback can be questioned [11, 12], which may lead to a suboptimal learning process [7]. With the development of rehabilitation technologies, new opportunities for providing external feedback have emerged [13]. For example, wireless inertial motion sensors can be used to provide easy to understand and accurate feedback to the patient (e.g. via an avatar) [13, 14]. As such, sensor-based postural feedback might be more effective than conventional feedback for improving movement control, which in turn may enhance treatment effects.

Although movement control exercises are widely used in a variety of chronic pain populations, little is known about the influence of chronic pain on the capacity to learn new movement skills. From a theoretical perspective, it has been suggested that patients with CLBP might have a reduced motor learning capacity [15]. One of the reasons for this hypothesis is that LBP can negatively influence proprioceptive acuity, leading to impaired intrinsic feedback from the lumbar spine [16]. As a consequence, patients with LBP might have to rely more on external feedback and become more dependent on it [7]. In addition, pain demands attention and can distract patients from the movement task [17], which might in turn interfere with the learning process [15]. However, empirical evidence for a reduced motor learning capacity in patients with CLBP is currently lacking and the scantly available research in other chronic pain populations shows equivocal results [18, 19].

Therefore, the aims of this study were (1) to assess whether sensor-based feedback is more effective to improve lumbopelvic movement control compared to feedback from a mirror or no feedback in patients with chronic low back pain (CLBP), and (2) to evaluate whether patients with CLBP are equally capable of improving lumbopelvic movement control compared to healthy persons.

## Methods

### Design

A randomised controlled trial including healthy persons and patients with CLBP was conducted. Both groups of participants were randomised into three subgroups, each receiving a different type of feedback during the intervention, i.e. feedback from sensors, a mirror or no feedback (= control group). Randomisation was done with a computerised random sequence generator and allocation concealment was obtained by using sequentially numbered, sealed, opaque envelopes prepared by a person not further involved in the study.

The intervention consisted of a single exercise session during which participants practised a movement control task while receiving their assigned type of feedback. Movement control was assessed with lumbopelvic kinematics, which were obtained at baseline, during and immediately after the intervention.

### Participants

Participants were recruited at private physiotherapy and GP practices and via social media. To be included, all participants needed to be between 18 and 65 years old and patients had to be diagnosed with chronic non-specific LBP (> 3 months, ≥3 days/week). Exclusion criteria for all participants were: spinal surgery in the past, an underlying serious disease or a physical problem interfering with daily life activities (e.g. severe knee pain), signs or symptoms of nerve root involvement, performance of lumbopelvic movement control exercises in the past year and pregnancy. Healthy subjects were also excluded if they experienced LBP in the past year.

To ensure that participants were able to achieve an improvement in movement control, the performance on the baseline movement control tasks was an additional inclusion criterion. To be included, the maximal lumbar range of motion during the baseline movement control tasks had to exceed 10° (0° would be a perfect performance). Participants with less range of motion on either of the baseline movement control tasks were excluded. Although this threshold of 10° was set a priori, the lumbar range of motion could only be calculated after completion of the full protocol. Therefore, all of the included participants completed the protocol, but only those fulfilling the abovementioned criterion were included in the final analysis.

### Assessments

#### Baseline assessments

**Fig. 1:**
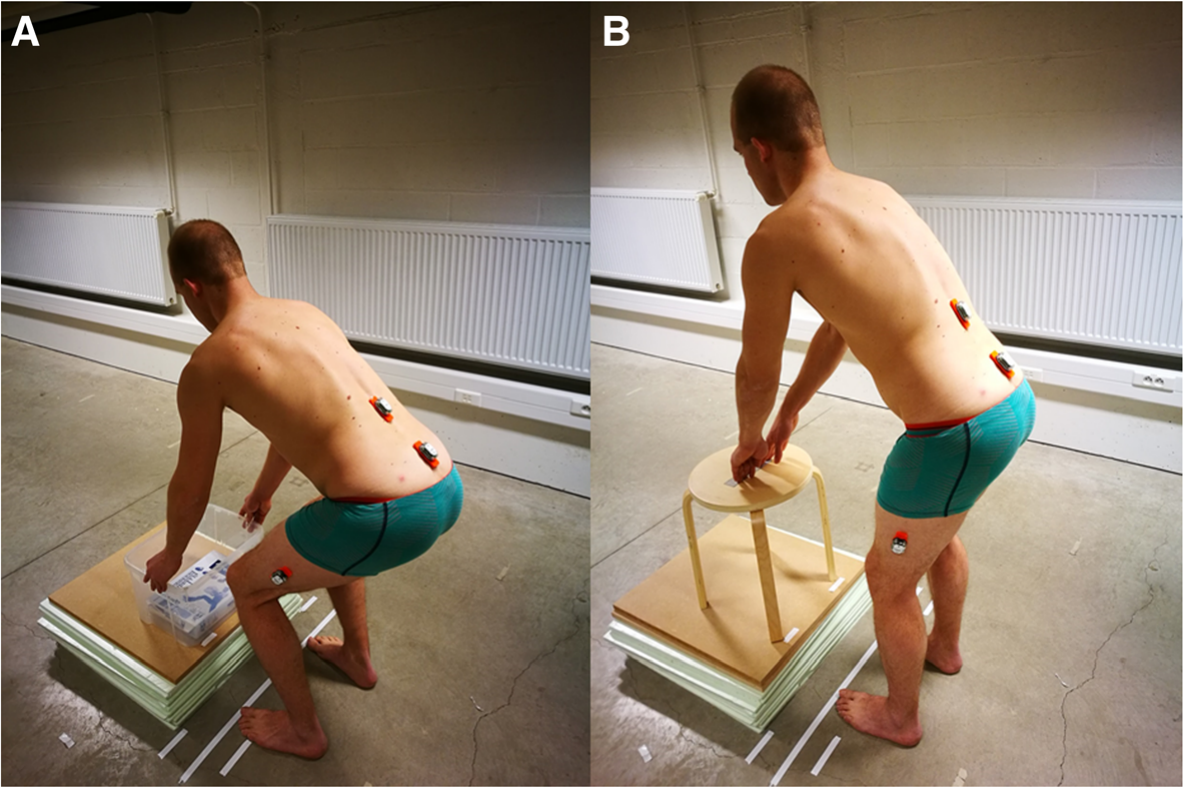
Movement control tasks. **a** Lifting task. **b** Waiter’s bow

Sociodemographic data were obtained from all participants. Patients with CLBP also completed the Numeric pain rating scale (NPRS) [20] to assess current pain and the average pain during the past 7 days, the Roland Morris Disability questionnaire (RMDQ) [21] to assess disability and the Tampa scale for kinesiophobia (Miller RP, Kori SH, Todd DD: The Tampa Scale, unpublished) to assess the fear of movement/re-injury due to physical activity. After completing the questionnaires, participants performed two movement control tasks, i.e. a lifting task followed by a waiter’s bow (Fig. 1). Both tasks were standardised for the participants’ height and assessed with lumbopelvic kinematic measurements in the sagittal plane. Before the baseline kinematic measurements, the tasks were explained and demonstrated in a standardised way. For the lifting task, participants started from a relaxed standing position and were asked to lift a box with handles from a platform on the floor and to put it back down, while maintaining their lumbar curvature (i.e. not to flex or extend the lumbar spine). Participants were allowed to flex their knees as far as they wanted to. The distance from the box to the hallux was 15 cm. The dimensions of the box were 40 × 30 × 23.5 cm, and it weighed 4 kg. The top of the box was positioned 10 cm below the apex of the subjects’ patella. For the waiter’s bow, participants started with slightly flexed knees (±20°). Participants were instructed to keep their knees in the same position and to bend forward in the hips while maintaining their lumbar curvature. Participants had to touch the middle of a stool, marked with a piece of tape, which was positioned 15 cm in front of the hallux, and to return to their starting position. No familiarisation was allowed, and each task was performed five times at a self-selected speed.

#### Assessments during and after the intervention

Kinematics were also obtained during and three minutes after the intervention. For the post-intervention kinematic assessment, participants first performed the waiter’s bow and then the lifting task as described above. Immediately after the post-intervention kinematic assessment, all participants were asked to complete the Borg-scale for perceived exertion [22], and to answer two questions on a 0 to 10 numeric rating scale: ‘what was your average LBP intensity during the experiment?’ (0 = no pain at all, 10 = worst imaginable pain), ‘how fearful were you to damage your back?’ (0 = not fearful at all, 10 = extremely fearful). If significant between group differences would be present on the post-intervention questionnaires, these would be controlled for in the data analysis, as they might influence movement patterns [23–25].

#### Equipment

The Valedo®motion research tool (Hocoma, Switzerland) was used to assess the lumbopelvic kinematics and to provide feedback in the sensor groups. This system consists of a laptop and three wireless inertial measurement sensors, which contain a magnetometer, 3D-accelerometer and a 3D-gyroscope. The sensors were placed on the spinous process of L1 and S1, and 20 cm above the lateral femoral condyle (Fig. 1). All three sensors were used for the kinematic assessment, while only the L1 and S1 sensors were used to provide feedback in the sensor groups. Details on the kinematic data acquisition have been previously described [26].

### Intervention

During the intervention, participants practised the waiter’s bow during three sets of six repetitions while they received their assigned form of feedback. Each set of exercises was separated by one minute of rest. The lifting task was not practised. The feedback in the different groups was provided as follows:

#### Sensor group

**Fig. 2:**
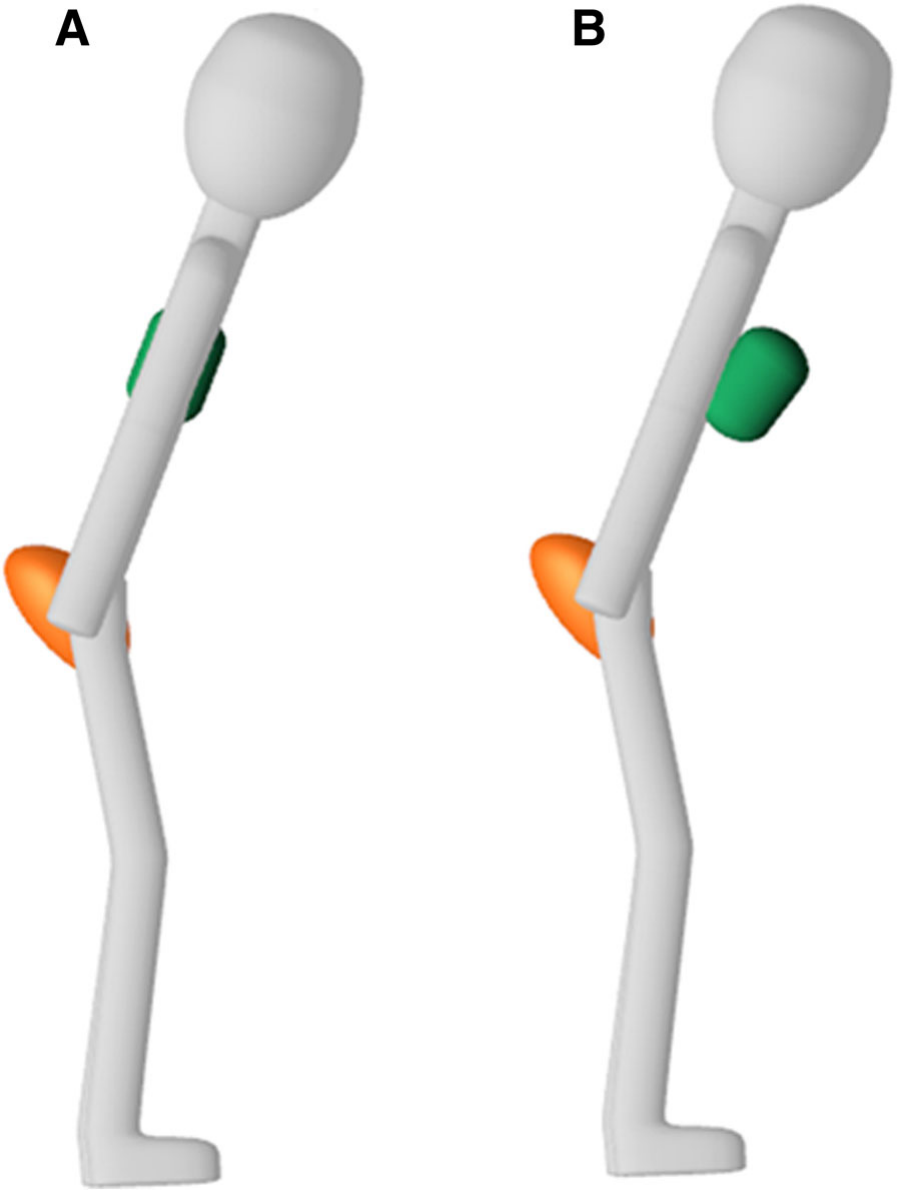
Sensor-feedback with an avatar. **a** The green rectangle is kept on the upper body of the avatar, indicating that the lumbar curvature is maintained. **b** The green rectangle moves anteriorly to the avatar’s upper body, indicating a lumbar flexion

The sensor-feedback was given via an avatar on a computer screen in front of the participants. The avatar was controlled by two movement sensors that were placed on the spinous process of L1 and S1. The upper body of the avatar corresponded with the S1-sensor and the green rectangle with the L1-sensor (Fig. 2). First, the system was calibrated when the participants assumed the starting position so that the green rectangle was placed in the middle of the avatar’s upper body. Participants were instructed to keep the green rectangle on the avatar during the exercises, as this meant that the lumbar curvature was maintained (Fig. 2a). If the rectangle moved anteriorly of the avatar, this corresponded with a lumbar flexion (Fig. 2b), while a posterior displacement indicated a lumbar extension.

#### Mirror group

A large mirror was placed laterally to the participants so they could see the stool and their whole body, and observe their spinal curvature during the exercises.

#### Control group

No feedback was provided.

ᅟ

Before the exercise trials, participants were explained how to use pelvic tilts to adjust the lumbar curvature. Hereafter, they were allowed to perform up to five pelvic tilts, during which participants in the sensor group could see how pelvic movements affected the position of the green rectangle relative to the avatar, participants in the mirror group could observe in the mirror how the pelvic tilts changed their lumbar curvature, while the control group received no feedback.

### Outcome measures for addressing the primary and secondary aims of the study

#### Primary aim - effectiveness of feedback

The influence of the different types of feedback on movement control performance and motor learning was of primary interest. Performance can be measured during or shortly after training, whereas motor learning can be assessed with a transfer test [27]. As the participants only practised the waiter’s bow, we used the differences between baseline and post-intervention kinematics of the waiter’s bow as a measure of performance, while differences in the lifting task kinematics were used as a measure for motor learning. For each repetition, the maximal range of motion in the lumbar spine and hip joint was calculated and expressed in absolute values. Lumbar spine angles were calculated from the L1 and S1 sensors, while hip joint angles were calculated from the S1 and femoral sensors. This method is highly reliable for both tasks in this study (ICCs = 0.89–0.93) [26]. The minimal detectable change between two measurements for the lifting task is 5.3° for the lumbar spine and 8.8° for the hip, while for the waiter’s bow this is 6.5° and 11.8°, respectively [26]. An improvement in movement control was defined as a decrease in the lumbar range of motion and an increase in the hip range of motion between baseline and post-intervention assessment. In addition to statistical significance, the abovementioned minimal detectable changes were used to interpret the results.

#### Secondary aim – Comparison between healthy persons and patients with CLBP

The differences between healthy subjects and patients with CLBP in movement control performance improvement and motor learning was evaluated. This was done by comparing the change in lumbopelvic kinematics between baseline and post-intervention between both participant groups. In addition, the evolution of the performance on the waiter’s bow task during the intervention was compared. In this way, it could be determined whether healthy participants and patients with CLBP needed the same number of repetitions to achieve an improvement on the waiter’s bow.

To investigate whether participants became dependent on the external feedback, the performance on the last exercise trial (with feedback) was compared with the post-intervention performance (without feedback) on the waiter’s bow. A significant decline on the post-intervention performance would indicate such dependence [7].

### Data analysis

The statistical analysis was performed with SAS JMP Pro (Version 12.2). To examine the effectiveness of the feedback and the difference in movement control improvement between healthy participants and patients with CLBP, a multiple linear regression was performed. The following variables were entered in the initial model to predict the differences between baseline and post-intervention kinematics: type of feedback (i.e. control, mirror or sensor), health status (i.e. healthy or CLBP), joint (i.e. lumbar spine or hip) and all their pairwise interactions. To control for the baseline values of the lumbar spine and hip angles, this variable was also put in the initial model. The variable ‘joint’ was included in the model because the movements of the spine and hip were considered to be related to each other. The final model was obtained by stepwise backward regression. The variable with the least significant *p*-value was left out first, and this was repeated until all the variables reached significance (*p* < 0.05). A Tukey all pairwise comparison was used as a post-hoc test.

A mixed model was used to assess the difference between healthy participants and patients with CLBP in the evolution of the performance on the waiter’s bow task. This model was also used to examine the difference between the last repetition of the intervention and the post-intervention performance on the waiter’s bow. The same variables from the linear regression were included in the mixed model, but ‘repetition number’ (i.e. baseline, repetitions during the intervention and post-intervention measurements were numbered) and its pairwise interactions with other variables were added as fixed factors. ‘Participant’ was used as a random factor to account for multiple measurements for the same participant.

Sample size calculation was based on an effect size (f^2^) of 0.2, power of 0.80 and α-level of 0.05. With these parameters, a total sample size of 80 participants was needed. Taking into account an attrition rate of 30% because of baseline performance on the movement control tests, 54 healthy persons and 54 patients with CLBP had to be recruited.

## Results

**Fig. 3:**
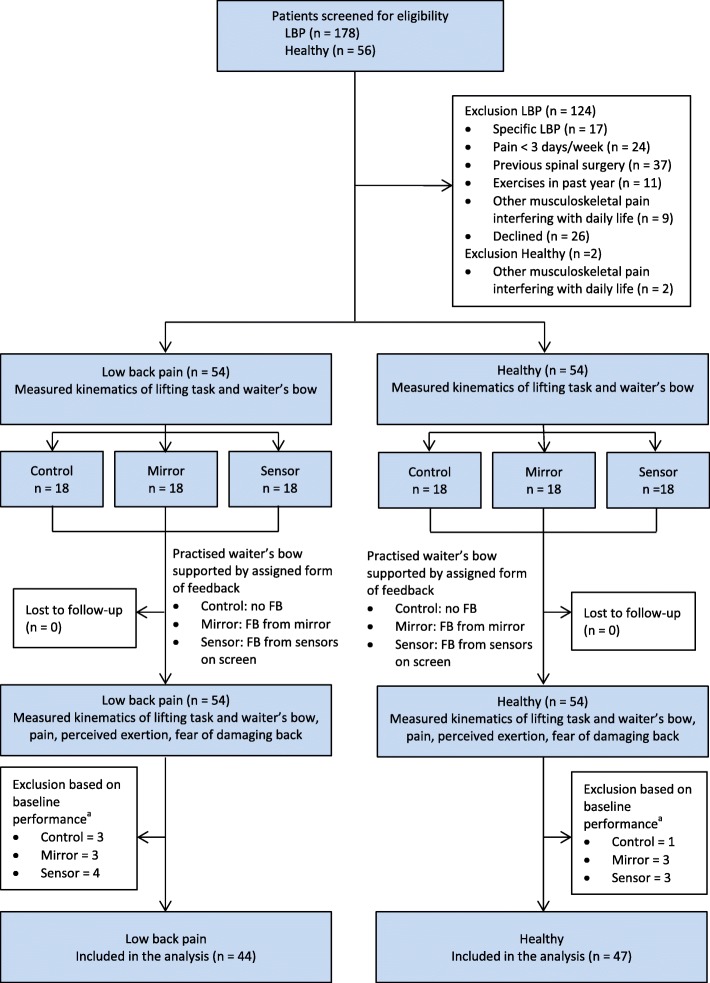
Design and flow of participants through the trial. FB = feedback. ^a^ Participants were excluded after the trial, based on their performance on the baseline movement control tasks (exclusion criterion set a priori). Because the performance on the baseline kinematic measurements was calculated after trial completion, all participants were measured post-intervention, but only 44 participants in the low back pain group and 47 participants in the healthy group were included in the final analysis

The flow of participants through the study is shown in Fig. 3. Ten (19%) patients with CLBP and seven (13%) healthy participants were excluded based on their baseline performance on the movement control tasks. No significant differences in demographics (Table 1) and baseline scores on kinematic outcome measures (Table 2, first column) were observed between groups.

**Table 1:** Baseline characteristics of the participants

Characteristic	Patients with chronic low back pain			Healthy persons						*p*-value
	Control (*n* = 15)	Mirror (*n* = 15)	Sensor (*n* = 14)	Control (*n* = 17)		Mirror (*n* = 15)		Sensor (*n* = 15)		
Sociodemographic data										
Age (years)	43 (12)	36 (13)	40 (17)	37 (10)		40 (14)		33 (14)		0.31
Gender, n female (%)	5 (33)	7 (47)	6 (43)	10 (59)		6 (40)		8 (53)		0.31
Height (cm)	176 (11)	175 (7)	171 (8)	174 (5)		170 (9)		172 (9)		0.38
Weight (kg)	78 (14)	69 (12)	70 (11)	70 (11)		63 (11)		71 (13)		0.05
LBP Questionnaires										
Onset LBP *(years)*^a^	3 (7)	4 (6)	6 (10)							0.56
NPRS 7 days *(0–10)*	4.9 (1.5)	4.5 (1.9)	4.5 (1.4)							0.72
NPRS current *(0–10)*	3.1 (2.0)	2.9 (1.9)	3.2 (2.2)							0.93
RMDQ *(0–24)*	7.7 (3.5)	7.5 (4.9)	6.6 (3.3)							0.69
TSK *(17–68)*	37.9 (5.5)	37.1 (6.9)	37.1 (8.6)							0.94

Data are mean (SD), unless mentioned otherwise

*LBP* low back pain, *NPRS* Numeric pain rating scale, *NPRS 7 days* average pain during the past 7 days measured with a NPRS, *NPRS current* current pain measured with a NPRS, *RMDQ* Roland-Morris Disability Questionnaire, *TSK* Tampa scale for kinesiophobia

^a^Median (IQR)

**Table 2:** Baseline and post-intervention maximal range of motion in the lumbar spine and hip joint

		Baseline	Post-intervention	Mean difference (95%CI)
Chronic low back pain				
Waiter’s bow				
Lumbar spine	Control	17.9 (5.9)	17.5 (6.6)	−0.4 (−2.9 to 2.0)
	Mirror	18.5 (4.3)	15.8 (2.7)	−2.7 (−0.5 to − 0.2)
	Sensor	16.2 (6.2)	6.5 (4.7)	− 9.7 (− 13.9 to − 5.5)^a^
Hip	Control	27.8 (16.3)	28.3 (15.8)	0.5 (− 4.7 to 5.8)
	Mirror	36.0 (13.7)	38.5 (14.2)	2.5 (− 3.4 to 8.4)
	Sensor	31.4 (9.8)	46.1 (11.8)	14.7 (6.4 to 23.0)^a^
Lifting task				
Lumbar spine	Control	23.7 (7.2)	22.0 (10.6)	− 1.7 (− 5.1 to 1.8)
	Mirror	20.5 (7.2)	18.9 (4.7)	− 1.6 (− 4.1 to 1.0)
	Sensor	21.0 (7.5)	13.9 (7.8)	−7.2 (− 3.7 to − 10.7)^a^
Hip	Control	89.2 (13.6)	87.3 (14.7)	− 1.9 (− 7.9 to 4.1)
	Mirror	91.1 (13.6)	86.3 (19.2)	− 4.9 (− 11.5 to 1.8)
	Sensor	89.7 (12.8)	95.4 (9.8)	5.7 (− 0.1 to 11.5)
Healthy subjects				
Waiter’s bow				
Lumbar spine	Control	20.5 (7.3)	18.7 (9.7)	−1.8 (−6.3 to 2.8)
	Mirror	22.2 (7.7)	20.6 (9.8)	−1.6 (−5.1 to 1.8)
	Sensor	21.5 (6.1)	8.2 (4.4)	− 13.3 (− 17.9 to − 9.4)^a^
Hip	Control	26.1 (10.5)	33.4 (13.8)	7.2 (− 1.6 to 12.9)
	Mirror	27.7 (12.7)	33.5 (15.1)	5.8 (1.1 to 10.4)
	Sensor	30.7 (10.1)	45.1 (7.4)	14.5 (9.2 to 19.7)^a^
Lifting task				
Lumbar spine	Control	24.1 (10.7)	22.4 (11.0)	− 1.8 (− 3.0 to − 0.7)
	Mirror	27.8 (7.0)	26.9 (7.3)	− 0.9 (− 3.7 to 1.8)
	Sensor	27.0 (8.3)	19.8 (7.0)	− 7.1 (− 2.6 to − 11.7)^a^
Hip	Control	88.0 (13.1)	86.7 (12.7)	− 1.3 (− 8.8 to 2.1)
	Mirror	92.4 (13.3)	92.6 (7.8)	0.2 (−4.2 to 4.6)
	Sensor	83.9 (14.1)	92.1 (10.7)	8.2 (3.1 to 13.3)

All data are expressed as angles in degrees (°). Data for baseline and post-intervention are mean (SD). Mean difference = post-intervention minus baseline

^a^Mean difference > measurement error

### Effectiveness of feedback

The results of the linear regression and post-hoc tests are presented in Table 3 (see Additional file 1 for a detailed sum of squares table). In both the healthy participants and patients with CLBP, the sensor group improved significantly more than the mirror and control group (post-hoc tests, *p* < 0.0001), while no differences were observed between the mirror and control group (post-hoc tests, *p* > 0.91). These results were obtained for both the waiter’s bow and the lifting task, as well as for the lumbar spine and hip. The improvements in the sensor groups were also larger than the measurement error (i.e. minimal detectable change), except for the hip during the lifting task (Table 2). There were no between groups differences in the post-intervention questionnaires (see Additional file 2).

**Table 3:** Results of the linear regression analysis and post-hoc tests for type of feedback

Linear regression		Post-hoc multiple comparisons for type of FB		
Fixed effects	*p*-value	Comparison	Estimated differences between groups (95% CI)	*p*-value
Waiter’s bow				
Initial model				
Health status	0.09			
Type of FB	< 0.0001			
Joint	0.01			
Baseline score kinematics	0.06			
Health status*type of FB	0.61			
Health status*Joint	0.71			
Type of FB*Joint	0.94			
Final model				
Type of FB	< 0.0001	Mirror minus Control	0.6 (− 3.1 to 4.4)	0.91
Joint	0.04	Sensor minus Control	10.6 (6.8 to 14.3)	< 0.0001^a^
		Sensor minus Mirror	9.9 (6.1 to 13.7)	< 0.0001^a^
Lifting task				
Initial model				
Health status	0.20			
Type of FB	< 0.0001			
Joint	0.029			
Baseline score kinematics	0.003			
Health status*type of FB	0.65			
Health status*Joint	0.44			
Type of FB*Joint	0.57			
Final model				
Type of FB	< 0.0001	Mirror minus Control	−0.3 (− 3.7 to 3.0)	0.97
Joint	0.02	Sensor minus Control	6.9 (3.5 to 10.2)	< 0.0001^a^
Baseline score kinematics	0.002	Sensor minus Mirror	7.2 (3.8 to 10.6)	< 0.0001^a^

*FB* Feedback, *Health status* healthy of CLBP, *Joint* lumbar spine or hip, *Type of FB* sensor, mirror or control

^a^in favour of the sensor group

Based on the type III sum of squares tables (see Additional file 1), it is clear that the type of feedback is the most important factor contributing to the variance that is explained by the final regression models of the waiter’s bow and lifting task, while the factor joint only explains a small proportion. A significant part of the variance that is explained by the final model of the lifting task can be attributed to the baseline scores on the kinematic assessments. Participants who had a worse performance on the baseline lifting task had a larger improvement.

### Comparison between healthy persons and patients with CLBP

**Fig. 4:**
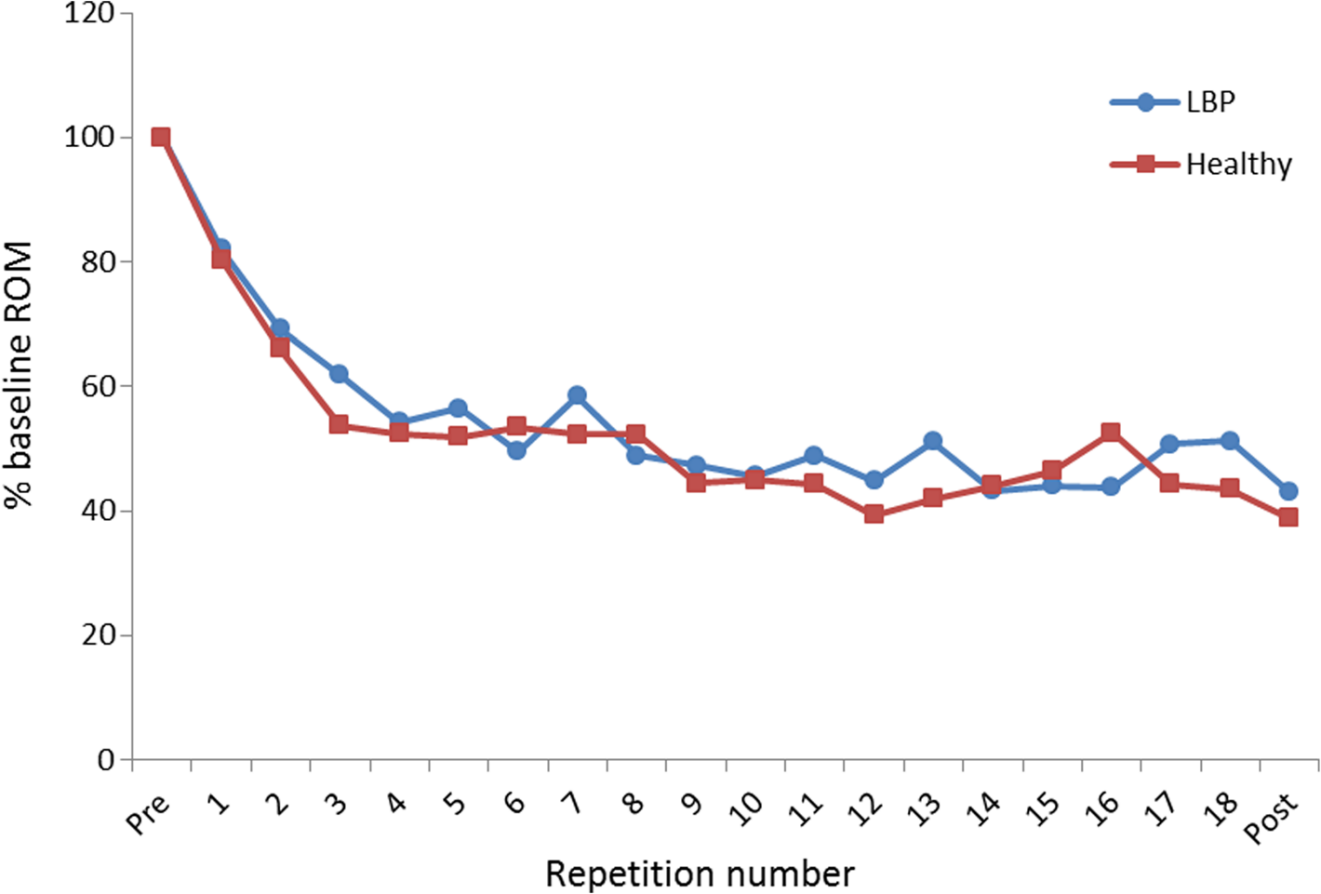
Evolution of the performance on the waiter’s bow in the Sensor groups throughout the intervention. On the Y-axis, the range of motion (ROM) in the lumbar spine is shown in proportion to the baseline ROM. A decrease in ROM indicates an improvement in movement control

The variable health status (i.e. healthy or CLBP) and its interaction with repetition number were not retained in the final mixed model (Table 4). This indicates that patients with CLBP were equally capable of improving lumbopelvic movement control, and that the evolution of the performance on the waiter’s bow task was similar between both participant groups (see Fig. 4 for an example of the sensor groups). These results are further supported by the fact that only a small proportion of the variance that is explained by the final model can be attributed to each of the variables pertaining to our second research question (see Additional file 3 for a detailed sum of squares table). Post-hoc tests also showed that there were no differences between the performance on the last exercise trial and the post-intervention assessment of the waiter’s bow. This demonstrates that participants in the mirror and sensor groups did not become dependent on the feedback.

**Table 4:** Results for the mixed model

Fixed effects	*p*-value
Initial model	
Health status	0.40
Type of FB	< 0.0001
Joint	< 0.0001
Baseline score kinematics	< 0.0001
Repetition number	< 0.0001
Health status*type of FB	0.83
Health status*Joint	0.01
Type of FB*Joint	0.08
Repetition number*type of FB	< 0.0001
Repetition number*Health status	0.28
Repetition number*Joint	0.09
Final model	
Health status	0.38
Type of FB	< 0.0001
Baseline score kinematics	< 0.0001
Joint	< 0.0001
Repetition number	< 0.0001
Health status*Joint	0.01
Repetition number*Type of FB	< 0.0001

*FB* Feedback, *Health status* healthy of CLBP, *Joint* lumbar spine or hip, *Type of FB* sensor, mirror or control

## Discussion

The primary aim of this study was to compare the effectiveness of different types of external feedback to improve lumbopelvic movement control in healthy persons and patients with CLBP. Our results show that sensor-based postural feedback was more effective to improve lumbopelvic movement control than feedback from a mirror or no feedback. Furthermore, being provided with feedback from a mirror did not lead to better results than receiving no feedback at all.

We hypothesize that the lack of improvement in the mirror group could be explained by the difficulty for unexperienced persons to visually detect changes in the lumbar curvature during the waiter’s bow. Although physiotherapists can reliably assess the waiter’s bow by observation, observer training may play an important role in this assessment [28]. Possibly, a longer teaching and familiarization period before the intervention could have enhanced the effectiveness of the mirror-feedback.

In contrast, the very short introduction to the sensor-feedback was sufficient to improve lumbopelvic movement control. We believe that the avatar provided more accurate and easy-to-understand feedback, which required no advanced training in order to interpret it correctly. It has been shown that abstract visualisations can be more effective than very realistic feedback (e.g. via a video or mirror) because they can provide information about key features of the task only, without overwhelming the participants with irrelevant information [8]. Participants in the sensor group only had to look at the green dot relative to the avatar’s upper body, while participants in the mirror group could also see movements in other body regions that were irrelevant to the task.

In addition, the screen displaying the avatar could be placed in front of the participants, whereas the mirror had to be positioned laterally to visualise the movements in the sagittal plane. Although this difference in position could be interpreted as a confounding factor because participants in the mirror group had to turn their heads in order to view their spinal curvature, the possibility to place the computer screen in the most convenient position should rather be considered as an inherent advantage of the sensor-feedback.

Finally, the improvements on the lifting task were partially explained by the baseline kinematic scores. Participants who performed worse on the lifting task at baseline assessment had a significantly larger improvement, which indicates that the motor learning effect was more pronounced in these participants. This might be explained by the fact that persons who performed worse at the baseline lifting task also had a larger potential for improvement.

Besides a mirror, various other types of conventional feedback, including tape or palpation [10], can be used to support patients during lumbopelvic movement control exercises. The rationale for comparing the sensor-based feedback to feedback from a mirror was twofold: First, a mirror is frequently being used or recommended to provide postural feedback during lumbopelvic movement control exercises [10, 29–31]. Second, and more importantly, both the mirror and sensors provided visual feedback, whereas palpation and a tape provide tactile feedback. Because visual motion detection is processed differently than tactile motion detection [32], we chose to compare the sensor feedback to feedback from a mirror.

Healthy subjects and patients with CLBP were equally capable of improving lumbopelvic movement control. It has been suggested that pain could negatively influence skill acquisition and motor learning by distracting people from the task they are performing [15]. However, this distraction mainly occurs when the pain is more intense, unfamiliar or unexpected [17]. The patients with CLBP in our study did not report an increase in pain during the exercise trials and there is no reason to assume that the pain they felt was unexpected or unfamiliar. Therefore, it is unlikely that patients with CLBP were distracted from the movement task.

Pain can also affect proprioceptive acuity and impair the intrinsic feedback system [33]. When less reliable intrinsic feedback is available, the dependency on the extrinsic feedback may increase [7]. Overall, patients with CLBP have decreased lumbosacral proprioception compared to healthy persons [16, 34], so it can be argued that removing the external feedback could influence the performance on the waiter’s bow more in patients with CLBP than in healthy participants. On the other hand, these proprioceptive impairments may be position specific (e.g. sit versus stance) [34] and little is known about proprioception during dynamic tasks [16], such as the ones in the present study. Our results show that omitting the external feedback had no influence on the performance on the waiter’s bow in both participant groups. This suggests that patients with CLBP also used information from the sensorimotor system to adjust their spinal curvature during the exercise trials [8], and that they did not rely more on the external feedback than healthy subjects. The improvements on the lifting task in the sensor groups further support this notion, as it indicates that both participant groups were able to transfer their newly learned skills to a different task. Therefore, it seems appropriate to use concurrent sensor-based feedback during the initial learning phase of movement control tasks in patients with CLBP.

Several limitations apply to this study. First, motor learning was only assessed with a transfer test, and not with a retention test. Both the transferability of practised skills and the long-term retention effects are important aspects of motor learning [27]. Because it is impossible to provide movement control training during every single activity an individual needs to perform, persons should be able to implement their newly acquired skills during activities that were not practised. In addition, the movement control improvements should be retained in the long term. However, because a retention test was not included in this study, we cannot make any statements regarding the longstanding effects of the sensor-based feedback.

Second, the mobility of the lower limb joints was not evaluated at baseline assessment. According to the concept of relative flexibility, a restriction in one joint could influence the movements in an adjacent joint [6]. Especially during the lifting task, more end range movements were necessary in the hip joint. As such, a restriction in hip joint mobility could have influenced the lumbar movements. On the other hand, participants with any physical problems other than LBP (e.g. hip or knee pain) that interfered with daily life activities were excluded from this study. Therefore, we believe it is unlikely that a (pathological) restriction of lower limb joint mobility would have significantly influenced the movement patterns in the lumbar spine.

Finally, our measurement and feedback system only contained three sensors. Due to these technical limitations, we could only measure the movements in the lumbar spine and hip joint. Consequently, we cannot exclude that some patients might have used compensatory movements in the thoracic spine while performing the movement control tasks. On the other hand, the reduction in lumbar ROM in the sensor group was accompanied by an increase in hip joint motion, indicating movements in the hip joint and lumbar spine were coupled.

## Conclusions

The recent development of rehabilitation technologies creates new possibilities for therapists and patients to support the rehabilitation process. As such, evaluating the effectiveness of these rapidly evolving technological systems poses an important challenge. The present study shows that sensor-based postural feedback is more effective than feedback from a mirror or no feedback to improve lumbopelvic movement control in the short term. Patients with CLBP were equally capable of improving lumbopelvic movement control as compared to healthy participants. Future research should focus on the long-term retention effects and evaluate whether supporting exercises with sensor-based feedback leads to larger improvements in pain and disability compared to conventional exercise therapy.

## Additional files

Additional file 1: Type III sum of squares table of the final model of the regression analyses. Table showing the Type III sum of squares table of the final model of the regression analyses. (DOCX 14 kb)

Additional file 2: Results for post-intervention questionnaires. Table showing the results for post-intervention questionnaires. (DOCX 15 kb)

Additional file 3: Type III sum of squares table for the mixed model analysis. Table showing the Type III sum of squares table for the mixed model analysis. (DOCX 14 kb)

## Abbreviations

CLBP: Chronic low back pain
LBP: Low back pain

## References

[1] Airaksinen O, Brox JI, Cedraschi C, Hildebrandt J, Klaber-Moffett J, Kovacs F, Mannion AF, Reis S, Staal JB, Ursin H, Zanoli G (2006). Chapter 4. European guidelines for the management of chronic nonspecific low back pain. Eur Spine J.

[2] Hurwitz EL, Randhawa K, Yu H, Cote P, Haldeman S. The global spine care initiative: a summary of the global burden of low back and neck pain studies. Eur Spine J. 2018. 10.1007/s00586-017-5432-9. 10.1007/s00586-017-5432-9 29480409

[3] Dagenais S, Caro J, Haldeman S (2008). A systematic review of low back pain cost of illness studies in the United States and internationally. Spine J.

[4] O'Sullivan P (2005). Diagnosis and classification of chronic low back pain disorders: maladaptive movement and motor control impairments as underlying mechanism. Man Ther.

[5] Luomajoki HA, Bonet Beltran MB, Careddu S, Bauer CM (2018). Effectiveness of movement control exercise on patients with non-specific low back pain and movement control impairment: a systematic review and meta-analysis. Musculoskeletal Science Practice.

[6] Sahrmann SA. Movement System Impairment Syndromes of the Extremities, Cervical and Thoracic Spines. 1st ed. St. Louis: Mosby; 2010.

[7] Magill RA (2007). Motor Learning and Control. Concepts and applications.

[8] Sigrist R, Rauter G, Riener R, Wolf P (2013). Augmented visual, auditory, haptic, and multimodal feedback in motor learning: a review. Psychon Bull Rev.

[9] Ribeiro DC, Sole G, Abbott JH, Milosavljevic S (2011). Extrinsic feedback and management of low back pain: a critical review of the literature. Man Ther.

[10] Hodges PW, Van Dillen LR, McGill SM, Brumagne S, Hides JA, Moseley GL, Hodges PW, Cholewicki J, Van Dieen JH (2013). Integrated clinical approach to motor control interventions in low back and pelvic pain. Spinal Control: The rehabilitation of back pain State of the art and science.

[11] Elgueta-Cancino E, Schabrun S, Danneels L, Hodges P (2014). A clinical test of lumbopelvic control: development and reliability of a clinical test of dissociation of lumbopelvic and thoracolumbar motion. Man Ther.

[12] Haneline MT, Cooperstein R, Young M, Birkeland K (2008). Spinal motion palpation: a comparison of studies that assessed intersegmental end feel vs excursion. J Manip Physiol Ther.

[13] Wang Q, Markopoulos P, Yu B, Chen W, Timmermans A (2017). Interactive wearable systems for upper body rehabilitation: a systematic review. J Neuroeng Rehabil.

[14] Matheve Thomas, Claes Guido, Olivieri Enzo, Timmermans Annick (2018). Serious Gaming to Support Exercise Therapy for Patients with Chronic Nonspecific Low Back Pain: A Feasibility Study. Games for Health Journal.

[15] Boudreau SA, Farina D, Falla D (2010). The role of motor learning and neuroplasticity in designing rehabilitation approaches for musculoskeletal pain disorders. Man Ther.

[16] Brumagne S, Janssens L, Claeys K, Pijnenburg M. Altered variability in proprioceptive control strategy in people with recurrent low back pain. In: Hodges P, Cholewicki J, Van Dieën JH, editors. Spinal control: The rehabilitation of back pain. 1st ed. London: Churchill Livingstone; 2013. p. 135–44.

[17] Eccleston C, Crombez G (1999). Pain demands attention: a cognitive-affective model of the interruptive function of pain. Psychol Bull.

[18] Vallence AM, Smith A, Tabor A, Rolan PE, Ridding MC (2013). Chronic tension-type headache is associated with impaired motor learning. Cephalalgia.

[19] Parker RS, Lewis GN, Rice DA, McNair PJ (2017). The association between Corticomotor excitability and motor skill learning in people with painful hand arthritis. Clin J Pain.

[20] Chapman JR, Norvell DC, Hermsmeyer JT, Bransford RJ, DeVine J, McGirt MJ, Lee MJ (2011). Evaluating common outcomes for measuring treatment success for chronic low back pain. Spine (Phila Pa 1976).

[21] Roland M, Morris R (1983). A study of the natural history of back pain. Part I: development of a reliable and sensitive measure of disability in low-back pain. Spine (Phila Pa 1976).

[22] Borg GA (1982). Psychophysical bases of perceived exertion. Med Sci Sports Exerc.

[23] Vaisy M, Gizzi L, Petzke F, Consmuller T, Pfingsten M, Falla D (2015). Measurement of lumbar spine functional movement in low back pain. Clin J Pain.

[24] Trost Z, France CR, Thomas JS (2009). Examination of the photograph series of daily activities (PHODA) scale in chronic low back pain patients with high and low kinesiophobia. Pain.

[25] van Dieen JH, van der Burg P, Raaijmakers TA, Toussaint HM (1998). Effects of repetitive lifting on kinematics: inadequate anticipatory control or adaptive changes?. J Mot Behav.

[26] Matheve T, De Baets L, Rast F, Bauer C, Timmermans A (2018). Within/between-session reliability and agreement of lumbopelvic kinematics in the sagittal plane during functional movement control tasks in healthy persons. Musculoskelet Sci Pract.

[27] Soderstrom NC, Bjork RA (2015). Learning versus performance: an integrative review. Perspect Psychol Sci.

[28] Carlsson H, Rasmussen-Barr E (2013). Clinical screening tests for assessing movement control in non-specific low-back pain. A systematic review of intra- and inter-observer reliability studies. Man Ther.

[29] Vibe Fersum K, O'Sullivan P, Skouen JS, Smith A, Kvale A (2013). Efficacy of classification-based cognitive functional therapy in patients with non-specific chronic low back pain: a randomized controlled trial. Eur J Pain.

[30] O'Sullivan PB, Caneiro JP, O'Keeffe M, Smith A, Dankaerts W, Fersum K, O'Sullivan K (2018). Cognitive functional therapy: an integrated behavioral approach for the targeted Management of Disabling low Back Pain. Phys Ther.

[31] Sheeran L, van Deursen R, Caterson B, Sparkes V (2013). Classification-guided versus generalized postural intervention in subgroups of nonspecific chronic low back pain: a pragmatic randomized controlled study. Spine (Phila Pa 1976).

[32] Nakashita S, Saito DN, Kochiyama T, Honda M, Tanabe HC, Sadato N (2008). Tactile-visual integration in the posterior parietal cortex: a functional magnetic resonance imaging study. Brain Res Bull.

[33] Roijezon U, Clark NC, Treleaven J (2015). Proprioception in musculoskeletal rehabilitation. Part 1: basic science and principles of assessment and clinical interventions. Man Ther.

[34] Tong MH, Mousavi SJ, Kiers H, Ferreira P, Refshauge K, van Dieen J (2017). Is there a relationship between lumbar proprioception and low back pain? A systematic review with meta-analysis. Arch Phys Med Rehabil.

