# Supplementary material for: Sensor-based postural feedback is more effective than conventional feedback to improve lumbopelvic movement control in patients with chronic low back pain: a randomised controlled trial
Source: J Neuroeng Rehabil. 2018 Sep 26;15:85. doi: 10.1186/s12984-018-0423-6 (PMC6156867; doi:10.1186/s12984-018-0423-6)
Supplement: Supplementary file 1 — Type III sum of squares table of the final model of the regression analyses. Table showing the Type III sum of squares table of the final model of the regression analyses. (DOCX 14 kb) [file 12984_2018_423_MOESM1_ESM.docx]

| **Additional file 1** Type III sum of squares table of the final models of the regression analyses | | | | | | | |
| --- | --- | --- | --- | --- | --- | --- | --- |
|  |  | Nparm | DF | Sum Sq | Mean Sq | F Ratio | p-value |
| *Waiter’s bow* | | | | | | | |
|  | Type of FB | 2 | 2 | 4159.99 | 2079.99 | 26.90 | <0.0001 |
|  | Joint | 1 | 1 | 303.54 | 303.54 | 3.93 | 0.049 |
|  |  |  |  |  |  |  |  |
| *Lifting task* | | | | | | | |
|  | Type of FB | 2 | 2 | 1953.44 | 976.72 | 15.97 | <0.001 |
|  | Joint | 1 | 1 | 319.79 | 319.79 | 5.23 | 0.02 |
|  | Baseline score kinematics | 1 | 1 | 592.64 | 592.64 | 9.69 | 0.002 |

FB= feedback
