# Supplementary material for: Sensor-based postural feedback is more effective than conventional feedback to improve lumbopelvic movement control in patients with chronic low back pain: a randomised controlled trial
Source: J Neuroeng Rehabil. 2018 Sep 26;15:85. doi: 10.1186/s12984-018-0423-6 (PMC6156867; doi:10.1186/s12984-018-0423-6)
Supplement: Supplementary file 2 — Results for post-intervention questionnaires. Table showing the results for post-intervention questionnaires. (DOCX 15 kb) [file 12984_2018_423_MOESM2_ESM.docx]

| **Additional file 2** Results for post-intervention questionnaires | | | | | | | |  |
| --- | --- | --- | --- | --- | --- | --- | --- | --- |
|  | Patients with CLBP | | |  | Healthy persons | | | p-value |
|  | Control | Mirror | Sensor |  | Control | Mirror | Sensor |  |
| Pain (*0 to 10*) | 3  (2 to 4) | 2  (0 to 3) | 3  (0 to 5) |  | 0  (0 to 0.5) | 0  (0 to 0) | 0  (0 to 1) |  |
| Pain difference *(-10 to 10)* | 0  (-2 to 0) | 1  (-1 to 2) | 0  (-0.5 to 1) |  | 0  (-0.5 to 0) | 0  (0 to 0) | 0  (-1 to 0) | 0.32 |
| Fear of damage *(0 to 10)* | 0  (0 to 1) | 0  (0 to 1) | 0  (0 to 1) |  | 0  (0 to 0) | 0  (0 to 0) | 0  (0 to 0) | 0.18 |
| Borg scale *(6 to 20)* | 9  (7 to 11) | 8  (7 to 12) | 9  (7 to 10) |  | 7  (6 to 8) | 6  (6 to 8) | 9  (6 to 11) | 0.10 |
| Data are median (IQR). Fear= fear of damaging the lumbar spine, Pain= average pain intensity during the intervention, Pain difference= pain baseline minus average pain during the intervention (a negative value indicates an increase in pain). | | | | | | | | |
