# Supplementary material for: Sensor-based postural feedback is more effective than conventional feedback to improve lumbopelvic movement control in patients with chronic low back pain: a randomised controlled trial
Source: J Neuroeng Rehabil. 2018 Sep 26;15:85. doi: 10.1186/s12984-018-0423-6 (PMC6156867; doi:10.1186/s12984-018-0423-6)
Supplement: Supplementary file 3 — Type III sum of squares table for the mixed model analysis. Table showing the Type III sum of squares table for the mixed model analysis. (DOCX 14 kb) [file 12984_2018_423_MOESM3_ESM.docx]

| **Additional file 3** Type III sum of squares table for the mixed model analysis | | | | | | |
| --- | --- | --- | --- | --- | --- | --- |
|  | NumDF | DenDF | Sum Sq | Mean Sq | F-value | p-value |
| Health status | 1 | 87.0 | 32.9 | 32.9 | 0.79 | 0.38 |
| Type of FB | 2 | 87.0 | 1213.4 | 606.7 | 14.57 | <0.0001 |
| Baseline score kinematics | 1 | 3551.5 | 8813.3 | 8813.3 | 211.69 | <0.0001 |
| Joint | 1 | 3510.6 | 15455.1 | 15455.1 | 371.24 | <0.0001 |
| Repetition number | 19 | 3488.0 | 6707.0 | 353.0 | 8.47 | <0.0001 |
| Health status*joint | 1 | 3492.5 | 247.5 | 247.5 | 5.94 | 0.01 |
| Repetition number*Type of FB | 38 | 3488.0 | 5545.8 | 145.9 | 3.51 | <0.0001 |
| FB= feedback | | | | | | |
